# Genetic factors have a major effect on growth, number of vertebrae and otolith shape in Atlantic herring (*Clupea harengus*)

**DOI:** 10.1371/journal.pone.0190995

**Published:** 2018-01-11

**Authors:** Florian Berg, Oda W. Almeland, Julie Skadal, Aril Slotte, Leif Andersson, Arild Folkvord

**Affiliations:** 1 University of Bergen, Department of Biology, Bergen, Norway; 2 Institute of Marine Research (IMR), Nordnes, Bergen, Norway; 3 Science for Life Laboratory, Department of Medical Biochemistry and Microbiology, Uppsala University, Uppsala, Sweden; 4 Department of Animal Breeding and Genetics, Swedish University of Agricultural Sciences, Uppsala, Sweden; 5 Department of Veterinary Integrative Biosciences, Texas A&M University, College Station, Texas, United States of America; Department of Agriculture and Water Resources, AUSTRALIA

## Abstract

Atlantic herring, *Clupea harengus*, have complex population structures. Mixing of populations is known, but the extent of connectivity is still unclear. Phenotypic plasticity results in divergent phenotypes in response to environmental factors. A marked salinity gradient occurs from Atlantic Ocean (salinity 35) into the Baltic Sea (salinity range 2–12). Herring from both habitats display phenotypic and genetic variability. To explore how genetic factors and salinity influence phenotypic traits like growth, number of vertebrae and otolith shape an experimental population consisting of Atlantic purebreds and Atlantic/Baltic F1 hybrids were incubated and co-reared at two different salinities, 16 and 35, for three years. The F1-generation was repeatedly sampled to evaluate temporal variation. A von Bertalanffy growth model indicated that reared Atlantic purebreds had a higher maximum length (26.2 cm) than Atlantic/Baltic hybrids (24.8 cm) at salinity 35, but not at salinity 16 (25.0 and 24.8 cm, respectively). In contrast, Atlantic/Baltic hybrids achieved larger size-at-age than the wild caught Baltic parental group. Mean vertebral counts and otolith aspect ratios were higher for reared Atlantic purebreds than Atlantic/Baltic hybrids, consistent with the differences between parental groups. There were no significant differences in vertebral counts and otolith aspect ratios between herring with the same genotype but raised in different salinities. A Canonical Analysis of Principal Coordinates was applied to analyze the variation in wavelet coefficients that described otolith shape. The first discriminating axis identified the differences between Atlantic purebreds and Atlantic/Baltic hybrids, while the second axis represented salinity differences. Assigning otoliths based on genetic groups (Atlantic purebreds vs. Atlantic/Baltic hybrids) yielded higher classification success (~90%) than based on salinities (16 vs. 35; ~60%). Our results demonstrate that otolith shape and vertebral counts have a significant genetic component and are therefore useful for studies on population dynamics and connectivity.

## Introduction

Phenotypic plasticity is the ability to display different phenotypes in response to environmental factors [1]. It has become increasingly important as the basic concept to discriminate marine fish populations [2] since individuals of a population are assumed to live under specific environmental conditions. Traditionally, fish populations have been identified based on phenotypic traits, although the relative importance of genetic and environmental factors on the determination of those phenotypic traits is generally unclear [3–5]. Therefore, the genetic and/or environmental mechanisms regulating phenotypic traits used for identification of fish populations need to be clarified and defined.

As one of the ecologically and commercially most important fish species in the northeastern Atlantic, herring (*Clupea harengus*) has been a key species for studies of population structure. Iles and Sinclair [6] proposed that Atlantic herring have complex population structure and much effort has been spent to resolve this structure. Phenotypic traits like growth [7, 8], numbers of vertebrae [9, 10], otolith microstructure [11, 12], as well as otolith shape [13–15], have been used to investigate the population structure of herring. Genetic studies have also become important during recent years. While no or very limited genetic differentiation was initially found between populations [16, 17], genome-wide analyses revealed clear genetic differentiation among Atlantic herring populations, but primarily at loci underlying ecological adaptation [18, 19].

Environmental factors have a strong influence on many phenotypic traits in herring. For example, temperature affects growth [20] and otolith microstructure [21, 22], as well as the number of vertebrae and can act in combination with salinity [23, 24]. While growth and otolith microstructure can vary over time with temperature, the number of vertebrae will be determined once during metamorphosis based on the experienced environmental conditions [25]. In herring, there is also a co-variability between genetic differentiation and salinity [19, 26, 27].

A strong salinity gradient, highly correlated with the genetic differentiation of herring in this area [19], occurs throughout the Baltic Sea from the inner Bothnian Bay (salinity <6) to the opening near the fully marine North Sea/Atlantic Ocean (salinity 35). Further, this salinity gradient is associated with differences in phenotypic traits of herring inhabiting these two environments. Beside the salinity, the temperature is often examined as the main factor determining variation in phenotypic traits. However, the average temperature difference between the Atlantic and Baltic (S5 Fig) is relatively minor compared to the vast salinity variation and subject also to marked seasonal variations. In addition, the lower average temperature in the Baltic contradicts the common assumption of a negative correlation between the number of vertebrae and temperature [23].

To understand or resolve the genetic and environmental influences on phenotypic traits, we used offspring of two herring populations (Atlantic vs. Baltic) that were genetically different and living in contrasting salinities in a common garden rearing experiment. Common garden experiments are designed to rear offspring from different populations under identical environmental conditions. Both Atlantic purebreds and Atlantic/Baltic hybrids were reared under controlled conditions with fixed salinities of either 16 or 35. Our main objectives were to explore genetic and salinity influences on phenotypic traits like growth, number of vertebrae and otolith shape. Further, the experiment was conducted over a 3-year period to evaluate potential temporal variation in growth and otolith shape. Temporal variation in the number of vertebrae was tested to determine if it was subjected to selection.

## Material and methods

Spring spawning herring caught by gillnets on 21 May 2013 in the Atlantic, approximately 12 km west of Bergen, Norway (60°34'11.2"N 5°0'18.9"E) and Baltic, approximately 80 km northeast of Uppsala, Sweden (60°38'52.0"N 17°48'44.2"E) were used as parental fish in this study. Half of the eggs from one Atlantic female were fertilized and incubated on the day of capture with sperm of one Atlantic male; the other half was fertilized with sperm of one Baltic male. The Atlantic herring were 5 years of age, 30.5 cm (female) and 32.5 cm (male) in total length with 57 vertebrae. The Baltic male was 8 years of age, 20.5 cm in total length with 55 vertebrae. The age of herring was determined by counts of winter rings from otoliths. The experimental setup, including only one mother, was designed to avoid any environmental maternal effects. The parental herring were from a subset of samples representing typical Atlantic and Baltic populations that exhibited huge phenotypic differences between groups (S6 Fig) and have been genetically characterized confirming population-specific differences [19]. S1 Appendix provides further details about the parental groups.

The fertilization and rearing experiment was conducted under common garden conditions at salinities 16 and 35, with values fluctuating during incubation between 15–17 and 34–35, respectively. Water temperatures varied with seasons with an average of 9.12±0.73°C and 9.04±0.71°C at salinity 16 and 35, respectively (S5 Fig) and the light intensities fluctuated according to the seasonal and daily cycle in Bergen (60°N). Fifty percent hatching, defined as day 0, occurred on 5 June 2013. Atlantic purebreds and Atlantic/Baltic F1 hybrids, hereafter called purebreds and hybrids, were co-reared at salinity 16 and 35 in two replicated 1 m circular tanks, including in total 1000 larvae at an initial purebred/hybrid ratio of 1:2. Herring larvae were fed in excess, firstly with live natural zooplankton and cultured rotifers [28] and later with *Artemia* spp. (23 days post hatching = DPH), until feeding on formulated feed started (71 DPH). On 3 October (120 DPH), juveniles were transferred into two 3 m circular tanks, one with salinity 16 and one with salinity 35, where the herring were reared further for nearly 3 years until their first maturity. This experimental setup generated four groups (*Pop*, in statistical models) which can be distinguished genetically into purebreds and hybrids, as well as by salinity: H16 = hybrid at 16, H35 = hybrid at 35, P16 = purebred at 16 and P35 = purebred at 35.

During the three years, we sampled 690 otoliths out of 950 herring (Table 1). All sampled fish were measured to the nearest mm and fin clipped for DNA analysis, whereas the number of vertebrae was counted only for some samples (n = 522, Table 1). The DNA samples were used to identify post-mortem Atlantic/Baltic hybrids and Atlantic purebreds by genotyping a diagnostic SNP using a Custom TaqMan® Assay Design Tool where the Baltic male was homozygous C (cytosine), while the Atlantic male and female were homozygous T (thymine) at a specific SNP locus (scaffold95_175856_SNP00029). The following TaqMan probe was used to distinguish between hybrids and purebreds:

GCTTCTTTGTCAGCAGAGGCACAGACATTT[T/C]GTTACATAAGGAGATGCTGTGTCAGGTCAG

where T/C, among brackets is the SNP assayed.

**Table 1:** Total numbers of analyzed herring and otoliths (in brackets).

DPH	H16	P16	H35	P35	Sample
187	85 (73)	14 (13)	69 (61)	30 (26)	1*
297	36 (31)	4 (2)	31 (24)	19 (18)	2*
482	0	0	76 (24)	37 (19)	
524	0	0	56 (0)	34 (0)	
531	0	0	17 (0)	8 (0)	
618	27 (23)	3 (2)	16 (14)	14 (12)	3*
702	0	0	10 (8)	10 (8)	
861	11 (8)	1 (1)	19 (13)	12 (11)	4
960	7 (3)	1 (1)	23 (22)	9 (9)	4*
1055	0	0	16 (15)	14 (14)	
1079	0	0	31 (31)	8 (8)	
1098	33 (33)	5 (4)	38 (37)	14 (13)	5*
1106	23 (22)	8 (7)	18 (18)	12 (12)	5*
1120	17 (17)	4 (4)	16 (15)	14 (14)	5*
**Total**	**240 (210)**	**39 (34)**	**436 (282)**	**235 (164)**	

Samples from different sampling days (DPH = days post hatching) that were combined for otolith analyses were marked with identical numbers in the rightmost column.

* Number of vertebrae was also counted. H16 = hybrids at salinity 16, P16 = purebreds at salinity 16, H35 = hybrids at salinity 35, P35 = purebreds at salinity 35.

A digital image of each otolith was captured using a Leica MZ95 stereomicroscope and reflected light with a Nikon digital sight DS-U1 microscope camera using the software NIS-elements F (Version 2.3). Following the method by Libungan et al. [29], otolith images were read into the R software [30], and otolith shape outlines were collected from the images using the shapeR package [31]. A discrete wavelet transformation to equally spaced radii from the otolith centroid to the otolith outline was conducted to obtain wavelet coefficients (unitless). Hereafter, otolith shape refers to variation in the wavelet coefficients representing the otolith shape outline. An analysis of covariance (ANCOVA) was performed individually for each sample (Table 1) to determine the effect of fish length on the wavelet coefficients, as well as otolith length and width. Coefficients which showed an interaction between the four herring groups and total length were excluded from the analysis (S2 Table). In this study, fish length could also be used as a proxy for the growth rate, because all fish from a sample had the same age (days post hatching, DPH). Further, the remaining coefficients, as well as otolith length and width, were adjusted for allometric relationships with fish length applying the normalization technique of Lleonart et al. [32].

For all model fittings, full and complex models were used as starting references and simplified in cases of non-significance. Length-at-age data, used as a proxy for somatic growth of individual herring, were fitted to the von Bertalanffy growth model [33]:

$${TL}_{t}={L_{\infty }}_{Pop}(1-e^{-K(t-t_{0})})$$

where *TL*_*t*_ is the average length at age *t*, *L*_*∞*_ is the asymptotic maximum length of each of the four herring groups (*Pop*), *K* is the von Bertalanffy growth rate coefficient, i.e., the rate at which length approaches the maximum length asymptote and *t*_*0*_ is the intercept on the time axis.

The aspect ratio (otolith length/width) was calculated for comparison with the parental groups. Further ANOVA tests were used to evaluate the significance of genetic origin (*Gen*) and salinity (*Sal*) on otolith width (*OW*) or length (*OL*) at given age classes (*DPH* as factor):

$$OW\;or\;OL=\alpha +\beta_{1}\times DPH+\beta_{2}\times Gen+\beta_{3}\times Sal$$

Differences in the otolith aspect ratio (*AR*) and the mean number of vertebrae (*VS*) among genetic origin (*Gen*) and salinity (*Sal*) were tested using a one-way ANOVA:

$$AR=\alpha +\beta_{1}\times Gen+\beta_{2}\times Sal$$

$$VS=\alpha +\beta_{1}\times Gen$$

The initial starting model also included, in both cases, the age (*DPH*) as a predictor variable, but this was removed due to non-significance. Since the number of vertebrae is fixed during metamorphosis, temporal variation would indicate some kind of selection, either through sampling or mortality. Significant differences among the four herring groups were identified using Tukey-HSD tests. A significance level α = 0.05 was applied for all analyses and statistical tests.

For a subset of the sampling days, otoliths had been obtained from fish in both salinities. Only these otoliths were used for the otolith shape analyses to allow for comparisons across salinities. In some cases (sample 4 and 5), adjacent sampling days (DPH) were combined due to low numbers (Table 1). Those samples were taken within 100 days of each other, and none of the analyzed characteristics differed. The temporal development of otolith shape outlines and general differences among the groups were examined visually by plotting the mean otolith shape outline of each group reconstructed of the wavelet coefficients (S1 Fig). To investigate which region of the otolith shape outline contributed most to the differences between the four groups, mean wavelet coefficients and their standard deviation were plotted against the angle of the outline. Thereby, each mean wavelet coefficient indicates the variation of the otolith shape outline within their predefined region. Further, the correlation within each group along the outline was estimated with an intraclass correlation (ICC). Consequently, a combination of a high mean wavelet coefficient (>0.25) and a high ICC indicated the region along the otolith shape outline that differed most.

For statistical analysis to demonstrate the variation in otolith shape represented by wavelet coefficients, Canonical Analysis of Principal Coordinates (CAP) [34] followed by ANOVA-like permutation tests were applied with 2000 permutations used to assess the significance of constraints. The CAP and ANOVA-like permutation tests were only applied to otoliths from herring of age 187 DPH and 1108 DPH (Table 1) because these samples provided enough otoliths from all groups to ensure reliable results. Finally, the ordinations of group averages were examined with the shape descriptors along the first two canonical axes. Using the CAP and the ANOVA-like permutation tests, otolith shape was compared among the four herring groups with an overall test, as well as by applying comparison between salinity and genetic groups. In addition, salinity effects on the otolith shape were investigated in the absence of any genetic differences by comparing hybrids originating from salinity 16 and 35 in isolation and purebreds in isolation. The genetic signal was examined in the same way in the absence of any salinity differences.

To validate if otolith shape analysis can be used for assigning individual herring to a given group, we applied a linear discriminant analysis (LDA) to the standardized wavelet coefficients of sample 1 and 5. The classification success into salinities, genetics, as well as the four groups, was estimated using the leave-one-out cross-validation [35]. Thus, each otolith was removed individually from the dataset and assigned to one of the predefined groups.

### Ethics statement

Herring samples of the parental Atlantic population were caught with permission of the Directorate of Fisheries, Bergen, Norway. The parental Baltic herring were purchased from a local commercial fisherman. The common garden experiment and rearing of the F1-generation was approved by the Norwegian national animal ethics committee (Forsøksdyrutvalget–FOTS ID-5072).

## Results

**Fig 1:**
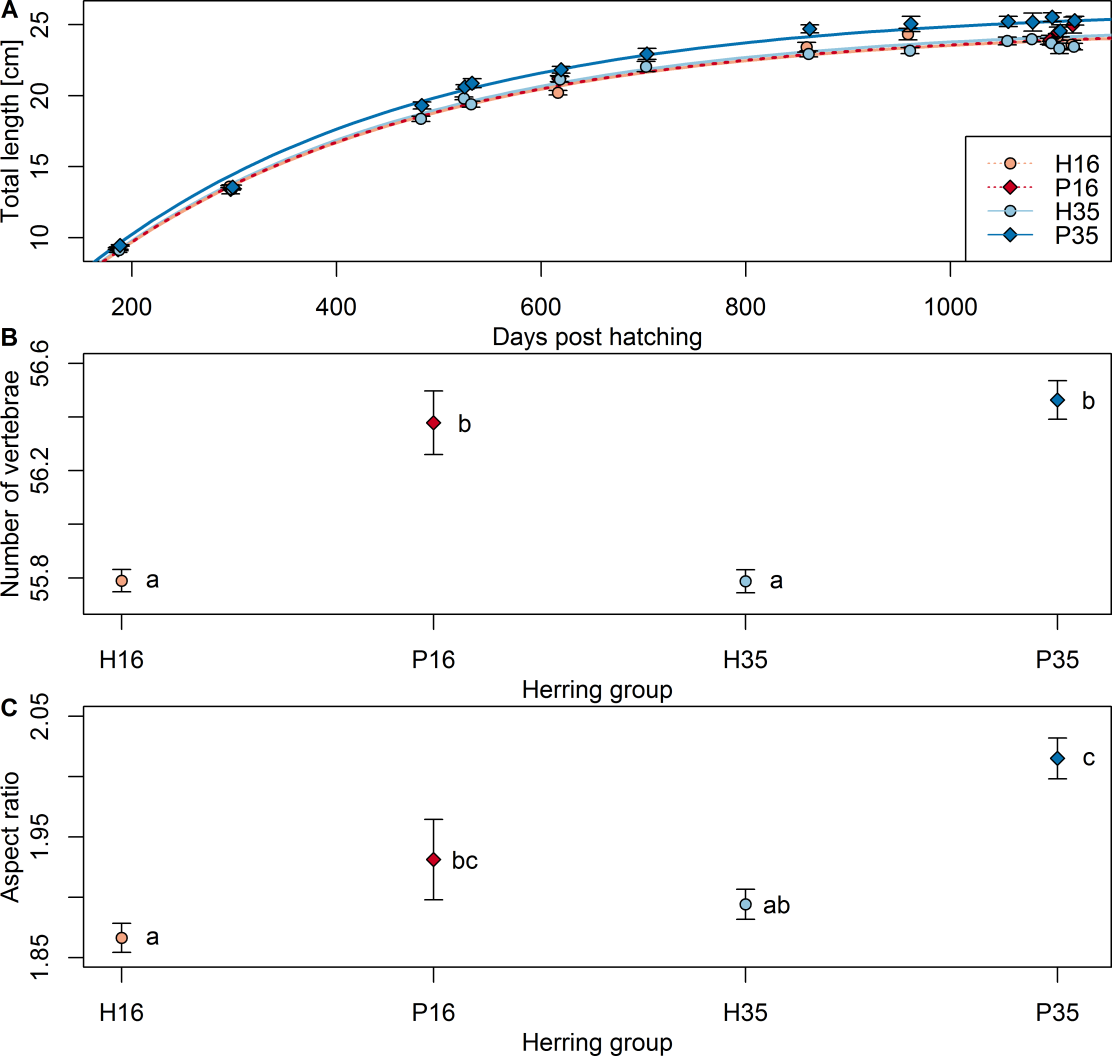
Comparison of phenotypic traits among the four herring groups. (A) length-at-age and von Bertalanffy growth models, (B) number of vertebrae, and (C) otolith aspect ratio were compared among H16 = hybrids at salinity 16, P16 = purebreds at salinity 16, H35 = hybrids at salinity 35, P35 = purebreds at salinity 35. Mean values and 1*SE are shown. Letters indicated posterior Tukey-HSD test results of all pair-wise comparisons. Groups which do not share a letter are significantly different to each other.

Somatic growth of herring reared under common garden conditions over three years only differed in the maximum asymptotic length among the four herring groups (Fig 1A). Atlantic purebreds reared at salinity 35 (*L*_*∞*_ = 26.2 cm) were larger (ANOVA: *F* = 194.5, *d*.*f*. = 944, *p*<0.001) than the other three groups (purebreds at salinity 16 (*L*_*∞*_ = 25.0 cm), Atlantic/Baltic hybrids at salinity 35 (*L*_*∞*_ = 24.8 cm) and 16 (*L*_*∞*_ = 24.8 cm)), which did not differ from each other (ANOVA: *F* = 0.69, *d*.*f*. = 712, *p*>0.05).

For the number of vertebrae, only a genetic effect could be demonstrated (Fig 1B; ANOVA: *F* = 109.1, *d*.*f*. = 520, *r*^*2*^ = 0.17, *p*<0.001). Mean vertebral counts were higher for Atlantic purebreds compared to Atlantic/Baltic hybrids irrespective of salinity (Tukey-HSD tests: *p*<0.001). The number of vertebrae did not differ over time within each group, indicating that no selection in terms of sampling or mortality had occurred for this trait (ANOVA: *F* = 0.97, *d*.*f*. = 517, *p*>0.05).

In general, otoliths of hybrids were shorter but wider compared with purebreds, but there were no differences between otoliths of herring originating from different salinities within each genetic group (Table 2). The otolith aspect ratio was higher for purebreds than hybrids (Fig 1C; ANOVA: *F* = 5.9, *d*.*f*. = 560, *r*^*2*^ = 0.08, *p*<0.001), and higher for herring reared at salinity 35 (ANOVA: *F* = 2.5, *p*<0.02). Purebreds at salinity 16 had a higher aspect ratio than hybrids at salinity 16, but the ratio was not significantly different from hybrids at salinity 35 (Tukey-HSD tests: *p*>0.05). The aspect ratio did not vary significantly between samples of different ages (ANOVA: *F* = 1.1, *d*.*f*. = 7, *p*>0.05). Additional results of the development of the otolith shape outline can be found in the S1 Appendix.

**Table 2:** Results from the ANOVA tests investigating the effects of age (days post hatching), salinity (16 vs. 35) and genetics (purebred vs. hybrid) on otolith width and length.

	Otolith width				Otolith length			
Variable	d.f.	MS	F	p	d.f.	MS	F	p
Days post hatching	11	23.46	3357.3	<0.001	11	105.09	4185.4	<0.001
Salinity	1	0.00	0.3	0.55	1	0.02	0.7	0.41
Genetics	1	0.10	13.6	<0.001	1	0.69	27.5	<0.001
Residuals	673	0.01			673	0.03		

d.f. = degrees of freedom, MS = mean square, F = *F*-value, p = *p*-value

**Fig 2:**
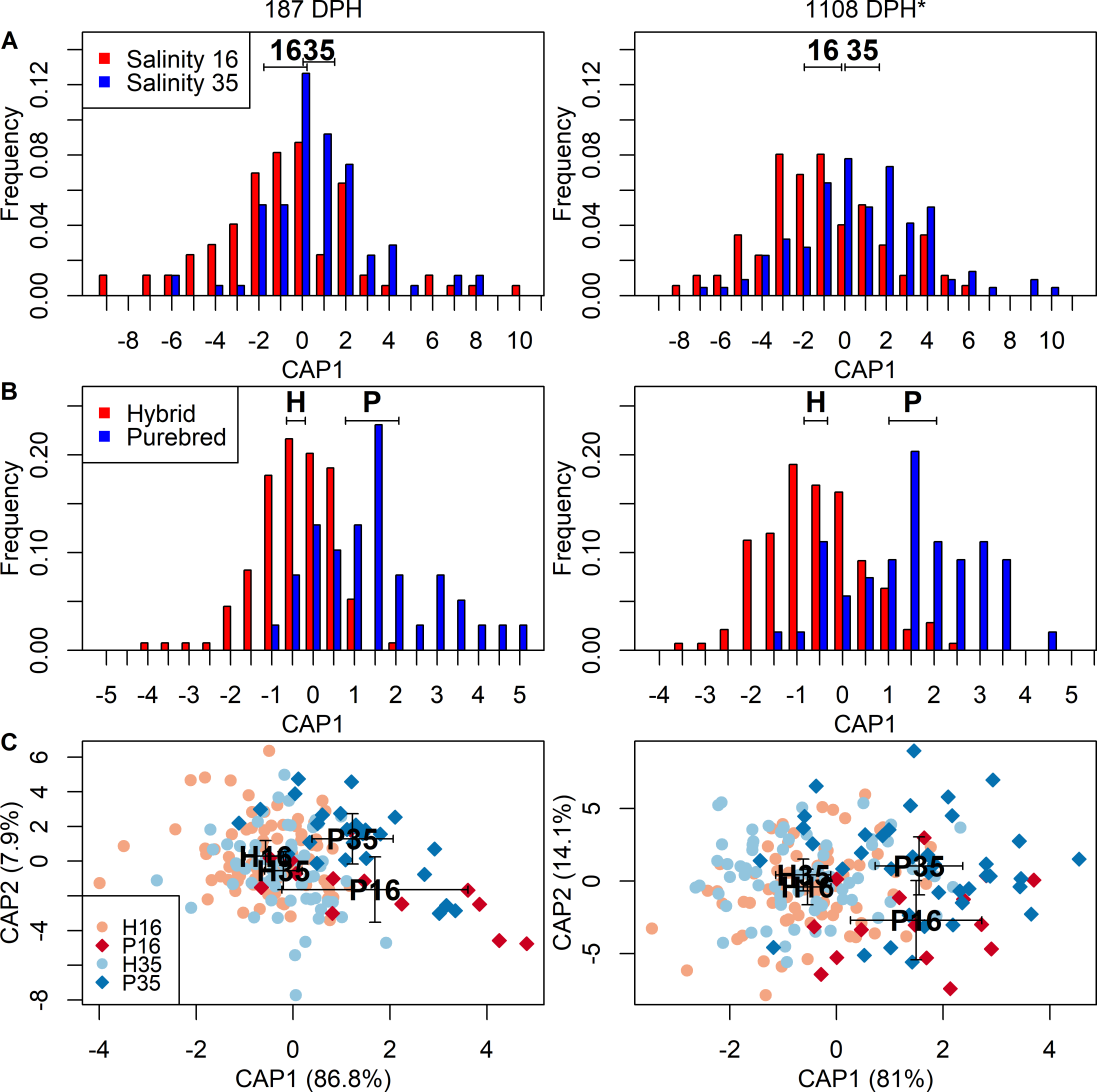
Canonical analysis of principal (CAP) scores of herring otolith shapes on discriminating axes. Scores of the first axis are shown for (A) salinity and (B) genetics, scores of the first and second axis for (C) all four groups. H16 = hybrids at salinity 16, P16 = purebreds at salinity 16, H35 = hybrids at salinity 35, P35 = purebreds at salinity 35. Black bold letters represent the mean canonical value for each character ± 1*SE. Individual fish are represented by frequencies (A, B) or symbols (C). * Mean day post hatching (DPH) for combined samples.

For the following analyses, only otoliths from herring at age 187 DPH and 1108 DPH were used due to a sufficient sample size. Significant differences in otolith shape, represented by wavelet coefficients, were observed between fish from the two salinities, between hybrids and purebreds, as well as among all four groups combined within both ages (Fig 2, Table 3). Comparing the salinity effect separated for hybrids and purebreds demonstrated significant differences (ANOVA: *F* = 2.0, *d*.*f*. = 1, *p* = 0.049) for hybrids from 187 DPH and purebreds from 1108 DPH, but not for purebreds from 187 DPH and hybrids from 1108 DPH (S3 Fig, S3 Table). While separating herring based on the salinity, otolith shape was always significantly different between hybrids and purebreds originating either from salinity 16 or 35 at both sampling days (S4 Fig). Genetic differences had a higher impact on the otolith shape than salinity differences, as indicated by higher *F*-values (Table 3). CAP and ANOVA-like permutation tests combining both parameters revealed clear differences among the four groups. The first canonical axis explained most of the variation between genetic groups, both for otoliths from 187 DPH (CAP1: 86.8%) and 1108 DPH (CAP1: 81.0%, Fig 2). Differences between the two salinities were explained by the second canonical axis, but only for otoliths from older herring (CAP2: 14.1%). The otolith shape of younger herring also varied along the second canonical axis, but not significantly and without any distinct pattern.

**Table 3:** Results from ANOVA-like permutation tests comparing the otolith shape (represented by wavelet coefficients) among salinities and genetic groups, as well as the four herring groups in the present study.

	187 days post hatching				1108 days post hatching			
Variable	d.f.	Var	F	p	d.f.	Var	F	p
Salinity	1	0.77	2.2	<0.05	1	1.87	2.3	0.03
Residuals	171	60.31			194	155.67		
Genetics	1	5.09	15.5	<0.001	1	11.89	15.8	<0.001
Residuals	171	55.99			194	145.65		
Genetics	1	5.09	15.7	<0.001	1	11.89	16.0	<0.001
Salinity	1	0.41	1.3	0.23	1	1.54	2.1	0.03
Genetics*Salinity	1	0.72	2.2	0.06	1	1.28	1.7	0.08
Residuals	169	54.87			192	142.83		

d.f. = degrees of freedom, Var = variance, F = *F*-value, p = *p*-value.

Otoliths from herring at age 187 DPH and 1108 DPH were classified based on their otolith shape. Classification success of otolith shape varied depending on the classifying character (Table 4). Otoliths of both ages had comparable results for each classifying character. Assigning otoliths based on salinities or the four groups had both a success rate between 50–60%, whereas based on the genetic groups ~90% of the otoliths were classified correctly. Splitting the results into individual comparisons (e.g., hybrids and purebreds when comparing the salinity effect) gave similar classification successes as for combined samples (Table 4).

**Table 4:** Overall classification success (bold) of otoliths into salinity, genetic groups and the four groups based on a linear discriminant analysis. The overall groups were evaluated separately (e.g. only hybrids were assigned to **salinity**) and their classification success presented. The analyses were conducted independently for otoliths from herring at different ages (187 and 1108 days post hatching = DPH).

	Classification success	
	187 DPH	1108 DPH
**Salinity**	**56.7%**	**60.7%**
Hybrid	58.2%	61.3%
Purebred	46.2%	55.6%
**Genetics**	**91.9%**	**87.2%**
Salinity 16	81.4%	77.0%
Salinity 35	81.6%	78.9%
**All groups**	**54.9%**	**49.5%**
Hybrid 16	58.9%	44.4%
Hybrid 35	54.1%	52.9%
Purebred 16	23.1%	33.3%
Purebred 35	61.5%	59.0%

## Discussion

This study provides the strongest evidence reported so far that the number of vertebrae and otolith shape (represented by wavelet coefficients) in Atlantic herring have a clear genetic basis and genetics had a more profound effect on these phenotypes than salinity. In general, this study confirms the genetic regulation of otolith shape [36]. The clearly demonstrated genetic effects based on phenotypic data from offspring were consistent with the phenotypic difference between the parental populations (Atlantic vs. Baltic herring). Further, temporal variations over a 3-year period in these traits were not evident, indicating that selection did not occur. The demonstrated differences in otolith aspect ratio or shape were genetically affected and independent of growth rate variations because the allometric relationship was removed by scaling the otoliths according to Lleonart et al. [32].

So far, most studies on population discrimination in fish have largely been based on phenotypic traits without knowledge of the specific genetic background influencing these traits (see references in [37]). Yet, studies combining genetic and environmental impacts are essential to understand population structures in marine fish. In this study, the genetic effect was the main factor underlying the observed phenotypic variation, supporting the use of genetic markers for population discrimination. Additional factors, such as temperature, could not be assessed with the current experimental design of common garden conditions. Further, phenotypes are not as informative as direct genetic data because a lack of phenotypic differences does not prove a lack of genetic differentiation.

Despite the large salinity differences experienced throughout the entire lifecycle of the experimental fish, salinity had only a minor impact on the phenotypic variation. However, long-term differences in population-specific salinity environments are not only considered to be associated with genetic distinctness [38–40] but can also lead to ecological speciation [41, 42]. Further, some fish species demonstrate higher growth at intermediate salinities than at a fully marine salinity [43, 44]. However, within this study Atlantic purebreds were growth retarded at salinity 16 compared with salinity 35, whereas the Atlantic/Baltic hybrids grew equally well in both salinities. This indicates the adaptation of Atlantic purebreds to high salinity. On the other hand, hybrids clearly outgrew the wild caught Baltic parental group within two years and had a much larger size-at-age (S6 and S7 Figs). It is possible that the genetic influence of the Atlantic parent contributed to this difference in growth conditions, but the captive F1-hybrids also grew at strikingly different environmental conditions compared with their wild-caught Baltic herring parent. In herring, some of the loci underlying genetic differentiation between Atlantic and Baltic herring show a strong correlation with average salinity conditions experienced by the different populations [18, 19]. However, there are also many other variables, for example in nutrition and temperature [21, 45], which could affect the growth of captive Atlantic/Baltic hybrids and wild Baltic herring. Herring in this study were fed in excess, and the water temperatures were generally higher than in the Baltic Sea (S5 Fig) which most likely promoted higher growth of Atlantic/Baltic hybrids compared to the Baltic parental group.

Besides salinity, temperature is known to have a high impact on phenotypic traits, like growth [46] and number of vertebrae [47]. Temperature has been demonstrated to be the major determinant of otolith growth, and therefore differences in otolith shape are essentially influenced by temperature [48, 49]. Further, environmental factors such as temperature and feeding conditions have impacts on otolith shape differences [14], even in the absence of genetic differences [50, 51]. However, in some cases, temperature does not affect otolith shape [52], or the genotype still has a higher impact than temperature on other phenotypic traits like growth [53, 54] or number of vertebrae [55]. Countergradient variation, i.e., the inverse relationship between environmental conditions and individual growth response [56], can maintain morphological similarity across populations and compensate for the effect of temperature [57].

Common garden experiments are ideally suited to dissect the relative importance of genetic and environmental factors affecting phenotypic traits [58, 59] and can play an essential role in resolving population structure [54]. Further, the exact knowledge of genetic and environmental origin is an enormous benefit, in contrast to natural samples where the origin can only be assumed. This knowledge was used for an individual assignment of otoliths, which achieved highest classification success when assigning the otoliths to their genetic origin (87.2–91.9%): this is comparable to other classification studies among populations using otolith shape [29, 60, 61]. Only a minor part of the otolith shape variation could be explained by salinity differences in the absence of genetic variation. Still, those differences are relevant for resolving the stock structure of herring, because otolith shape is also used to distinguish between groups that currently cannot be separated using genetics [62].

This high classification success based on otolith shape is practically used to separate populations from mixed fisheries (see, e.g., [60, 63]). This could then be incorporated in fisheries management and assessment to allow more sustainable exploitation of the populations [64]. Also in herring, otolith shape is used to discriminate between mixing stocks with varying spawning seasons [65]. However, there are other examples where spawning components have been identified but are still assessed as a unit stock [49]. Based on our results demonstrating the effect of genetics, we encourage the establishment of otolith shape baselines. These baselines can be further used in combination with machine-learning techniques [66] to assign individuals from mixed fisheries to populations.

In conclusion, our study revealed that the variation in several phenotypic traits, specifically growth, otolith shape and the number of vertebrae, was primarily controlled by genetic factors, while salinity played a minor role. To the best of our knowledge, this is the first time that hybrids of two herring populations were reared under common garden conditions until maturity. Finally, our results show that some of the phenotypic traits included in this study provide information to distinguish genetically differentiated herring. These phenotypic traits can be further used to study population dynamics and connectivity because they are to a large extent genetically determined. However, other factors, which were excluded due to the experimental design, might outplay the genetic response demonstrated within this study.

## Supporting information

S1 Appendix Further details on the otolith shape outline development, as well as the parental group. (PDF)

S1 Table Total numbers of analyzed parental fish and otoliths (in brackets) for each sample and parental group. (PDF)

S2 Table Number (N) and wavelet coefficients that were removed by adjusting otolith shape for allometric relationships with fish length individually for each sample. (PDF)

S3 Table Results from ANOVA like permutation tests comparing the otolith shape among salinities and genetic groups in isolation. (PDF)

S1 Fig An otolith shape outline example with lines indicating length and width going through the center of gravity. PoR = postrostrum, PaR = pararostrum, EMi = excisura minor, EMa = excisura major, R = rostrum, AR = antirostrum.
(TIF)

S2 Fig **(A) Mean otolith shape outline reconstructed from wavelet coefficients (unitless) and (B) their differences at respective otolith angle.** Data are shown for each sampling date and the four herring groups (H16 = hybrids at salinity 16, P16 = purebreds at salinity 16, H35 = hybrids at salinity 35, P35 = purebreds at salinity 35). The mean and standard deviation (SD) of the wavelet coefficients represent otolith shape outline variation among all groups and the intraclass correlation (ICC, black solid line) represents the variation within each group. * Mean day post hatching (DPH) for combined samples. Note that the otolith outline for P16 at 279, 618 and 910 DPH is based on N = 2.
(TIF)

S3 Fig Canonical analysis of principal (CAP) scores of herring otolith shapes indicating differences for salinity separated by the genetic groups (Hybrids and purebreds). Data given for the samples A) 187 and B) 1098 days post hatching. Black bold letters represent the mean canonical value for each character ± 1*SE. Individual fish are represented by frequencies.
(TIF)

S4 Fig Canonical analysis of principal (CAP) scores of herring otolith shapes indicating differences for genetics separated by salinity (16 and 35). Data given for the samples A) 187 and B) 1098 days post hatching. Black bold letters represent the mean canonical value for each character ± 1*SE. Individual fish are represented by frequencies.
(TIF)

S5 Fig Daily water temperatures Atlantic purebred and Atlantic/Baltic hybrids were reared at their entire life in either salinity 16 (light blue) or salinity 35 (dark blue). Water temperatures of the Atlantic (light red) were measured at stationary hydrographic stations in Ytre Utsira and Sognesjøen. Daily temperatures were combined for both stations and average for depths from 20–120 meters. Water temperatures of the Baltic (dark red) were extracted from https://sharkweb.smhi.se/ and restricted to the area 16–23° E and 56.5–62° N. Daily temperatures were combined all stations within the area and average for depths from 20–50 meters. Mean±SD are given in the legend and lines represent a running mean.
(TIF)

S6 Fig Weight-at-length data of the parental groups. Individuals used as parents for the F1-generation are marked (Atlantic male, Atlantic female, Baltic male).
(TIF)

S7 Fig Comparison of mean length (left), number of vertebrae (middle), and otolith aspect ratio (otolith length/otolith width, right) among the parental fish. Mean values and 1*SE are shown.
(TIF)

S8 Fig Average otolith shape outline for parental groups. The shown outline does not correspond to the actual size and ratio of the original otoliths.
(TIF)
