# Supplementary material for: Genetic factors have a major effect on growth, number of vertebrae and otolith shape in Atlantic herring (Clupea harengus)
Source: PLoS One. 2018 Jan 11;13(1):e0190995. doi: 10.1371/journal.pone.0190995 (PMC5764352; doi:10.1371/journal.pone.0190995)
Supplement: S1 Appendix — (PDF) [file pone.0190995.s001.pdf]

S1 Appendix

Results

Otolith shape outline development

The development of otolith shape outline (see S1 Fig for nomenclature) over three years was similar among the four groups (S2 Fig A). At the early stage (187 days post hatching), the *excisura major* was between the *rostrum* and the *antirostrum*, but with increasing age of herring, the *antirostrum* became more prominent and was more *anterior* than the *excisura major*. A similar development was observed with the *postrostrum* and *pararostrum*. At early ages, the *pararostrum* was more developed, changing to a more prominent *postrostrum* over time.

Otolith shape outline differed among all four groups, as visually reflected in mean shape differences (S2 Fig A) and a high level of variation in the wavelet coefficients among the groups (S2 Fig B). Depending on the age of the otolith the specific regions showing the highest variation among the groups in combination with high within groups correlation (ICC) differed, but in general the main differences were found along the otolith outline at 200-240° (S2 Fig B).

Parental group

Spring spawning herring caught 21st May 2013 in the Atlantic (60°34'11.2"N 5°0'18.9"E) and Baltic (60°38'52.0"N 17°48'44.2"E) were used as parental fish in this study. An additional sample of the parental groups was taken during the spring spawning season (S1 Table). The same methods and analysis as for the F1-generation were applied for the parental groups. There were no differences between the samples. The age of parental fish was not completely identified. Therefore, no length-at-age data is available and only mean length for each group is given. Instead weight-at-length data are given (S6 Fig) to demonstrate the phenotypic differences between the two parental groups. Individuals used for crossing out the F1-generation are marked. All parental fish were in spawning conditions and older than 3 years. The results for the parental groups are summarized in S7 and S8 Fig.
