# Supplementary material for: Genetic factors have a major effect on growth, number of vertebrae and otolith shape in Atlantic herring (Clupea harengus)
Source: PLoS One. 2018 Jan 11;13(1):e0190995. doi: 10.1371/journal.pone.0190995 (PMC5764352; doi:10.1371/journal.pone.0190995)
Supplement: S1 Table — (PDF) [file pone.0190995.s002.pdf]

S1 Table. Total numbers of analyzed parental fish and otoliths (in brackets) for each sample and parental group.

Date	Baltic	Atlantic
13.5.2013	42 (35)	9 (8)
21.5.2013	48 (47)	109 (63)
Total	90 (82)	118 (71)
