# Supplementary material for: Genetic factors have a major effect on growth, number of vertebrae and otolith shape in Atlantic herring (Clupea harengus)
Source: PLoS One. 2018 Jan 11;13(1):e0190995. doi: 10.1371/journal.pone.0190995 (PMC5764352; doi:10.1371/journal.pone.0190995)
Supplement: S2 Table — (PDF) [file pone.0190995.s003.pdf]

S2 Table. Number (N) and wavelet coefficients that were removed by adjusting otolith shape for allometric relationships with fish length individually for each sample.

Days post hatching	N	Removed coefficients
187	8	7, 8, 32, 34, 38, 44, 45, 54
297	2	56, 58
618	0	
910	4	8, 16, 53, 62
1108	0	
