# Supplementary material for: Genetic factors have a major effect on growth, number of vertebrae and otolith shape in Atlantic herring (Clupea harengus)
Source: PLoS One. 2018 Jan 11;13(1):e0190995. doi: 10.1371/journal.pone.0190995 (PMC5764352; doi:10.1371/journal.pone.0190995)
Supplement: S3 Table — (PDF) [file pone.0190995.s004.pdf]

S3 Table. Results from ANOVA like permutation tests comparing the otolith shape among salinities and genetic groups in isolation.

| | | **187 days post hatching** | | | | **1108 days post hatching** | | | |
|---|---|---|---|---|---|---|---|---|---|
| **Isolation factor** | **Variable** | **d.f.** | **Var** | **F** | **p** | **d.f.** | **Var** | **F** | **p** |
| **Hybrid** | Salinity | 1 | 0.59 | 2.0 | 0.049 | 1 | 0.92 | 1.4 | 0.174 |
| | Residuals | 132 | 38.85 | | | 140 | 92.64 | | |
| **Purebred** | Salinity | 1 | 0.54 | 1.2 | 0.237 | 1 | 1.90 | 2.0 | 0.049 |
| | Residuals | 37 | 16.01 | | | 52 | 50.19 | | |
| **Salinity 16** | Genetics | 1 | 3.18 | 9.0 | <0.001 | 1 | 4.52 | 6.5 | <0.001 |
| | Residuals | 84 | 29.63 | | | 85 | 59.19 | | |
| **Salinity 35** | Genetics | 1 | 2.27 | 7.6 | <0.001 | 1 | 8.32 | 10.6 | <0.001 |
| | Residuals | 85 | 25.23 | | | 107 | 83.64 | | |

d.f. = degrees of freedom, Var = variance, F = *F*-value, p = *p*-value.
