# Supplementary figures and images for: Genetic factors have a major effect on growth, number of vertebrae and otolith shape in Atlantic herring (Clupea harengus)
Source: PLoS One. 2018 Jan 11;13(1):e0190995. doi: 10.1371/journal.pone.0190995 (PMC5764352; doi:10.1371/journal.pone.0190995)

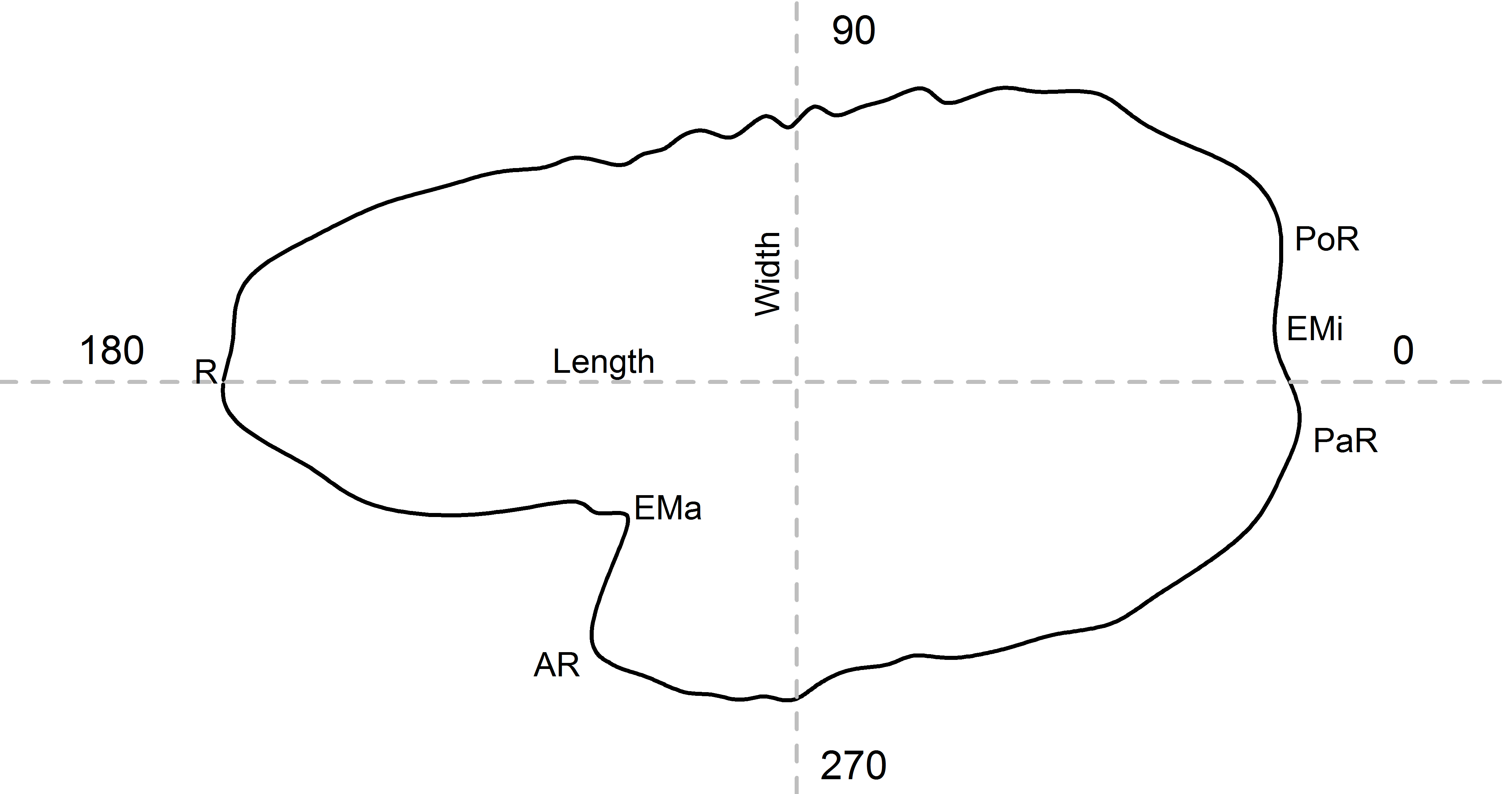

Supplement: S1 Fig — PoR = postrostrum, PaR = pararostrum, EMi = excisura minor, EMa = excisura major, R = rostrum, AR = antirostrum. (TIF) [file pone.0190995.s005.tif]

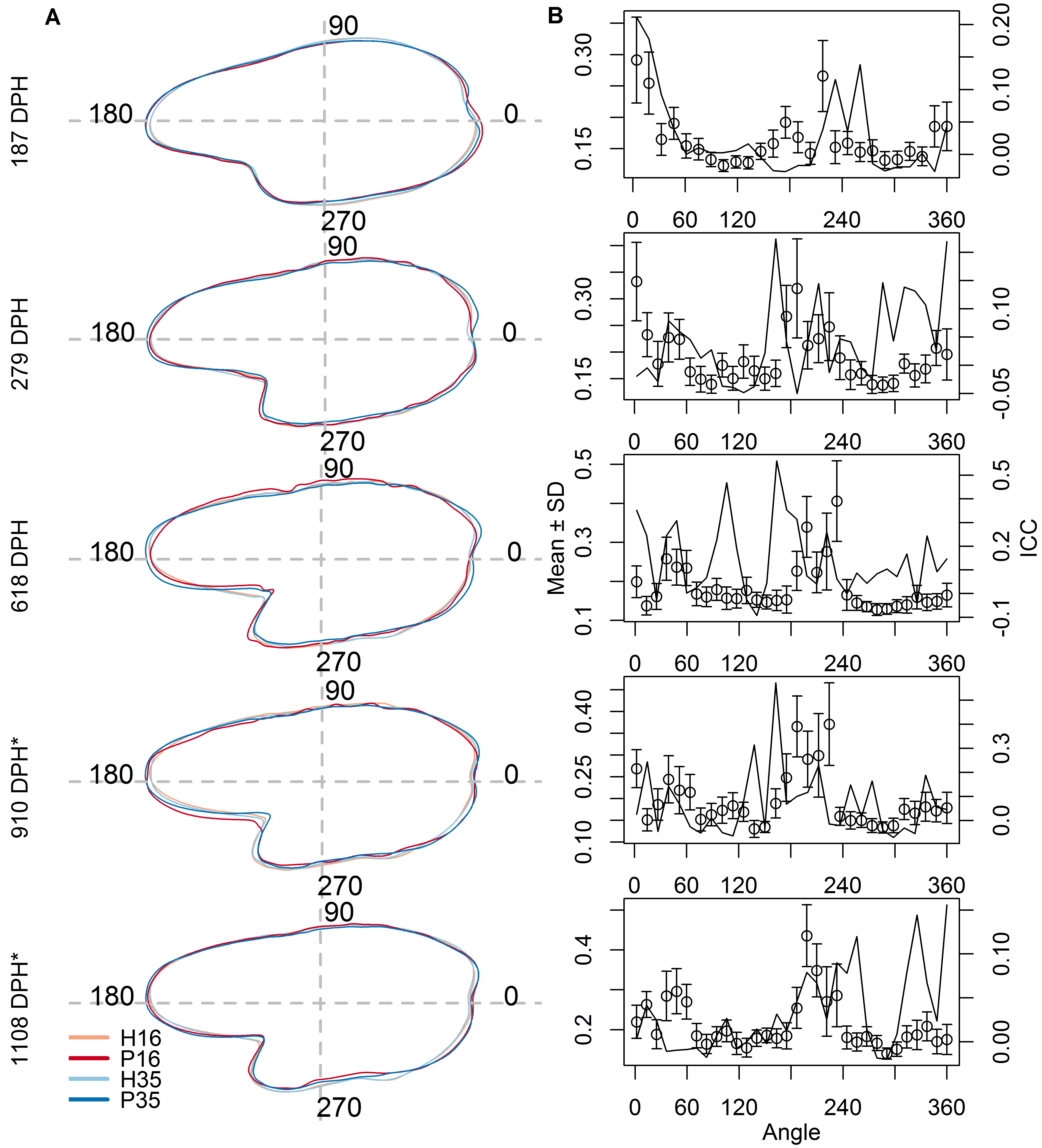

Supplement: S2 Fig — (A) Mean otolith shape outline reconstructed from wavelet coefficients (unitless) and (B) their differences at respective otolith angle. Data are shown for each sampling date and the four herring groups (H16 = hybrids at salinity 16, P16 = purebreds at salinity 16, H35 = hybrids at salinity 35, P35 = purebreds at salinity 35). The mean and standard deviation (SD) of the wavelet coefficients represent otolith shape outline variation among all groups and the intraclass correlation (ICC, black solid line) represents the variation within each group. * Mean day post hatching (DPH) for combined samples. Note that the otolith outline for P16 at 279, 618 and 910 DPH is based on N = 2. (TIF) [file pone.0190995.s006.tif]

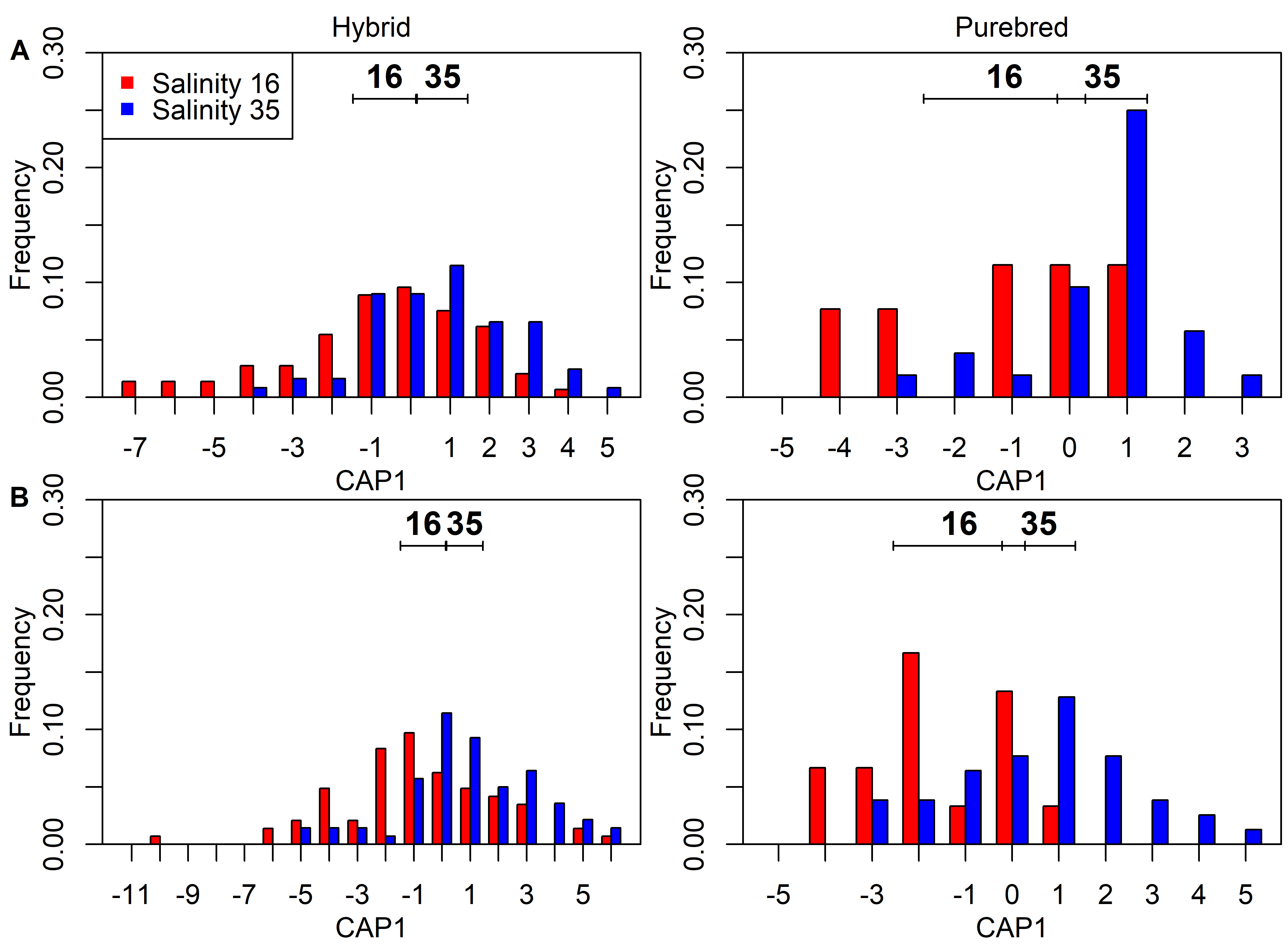

Supplement: S3 Fig — Data given for the samples A) 187 and B) 1098 days post hatching. Black bold letters represent the mean canonical value for each character ± 1*SE. Individual fish are represented by frequencies. (TIF) [file pone.0190995.s007.tif]

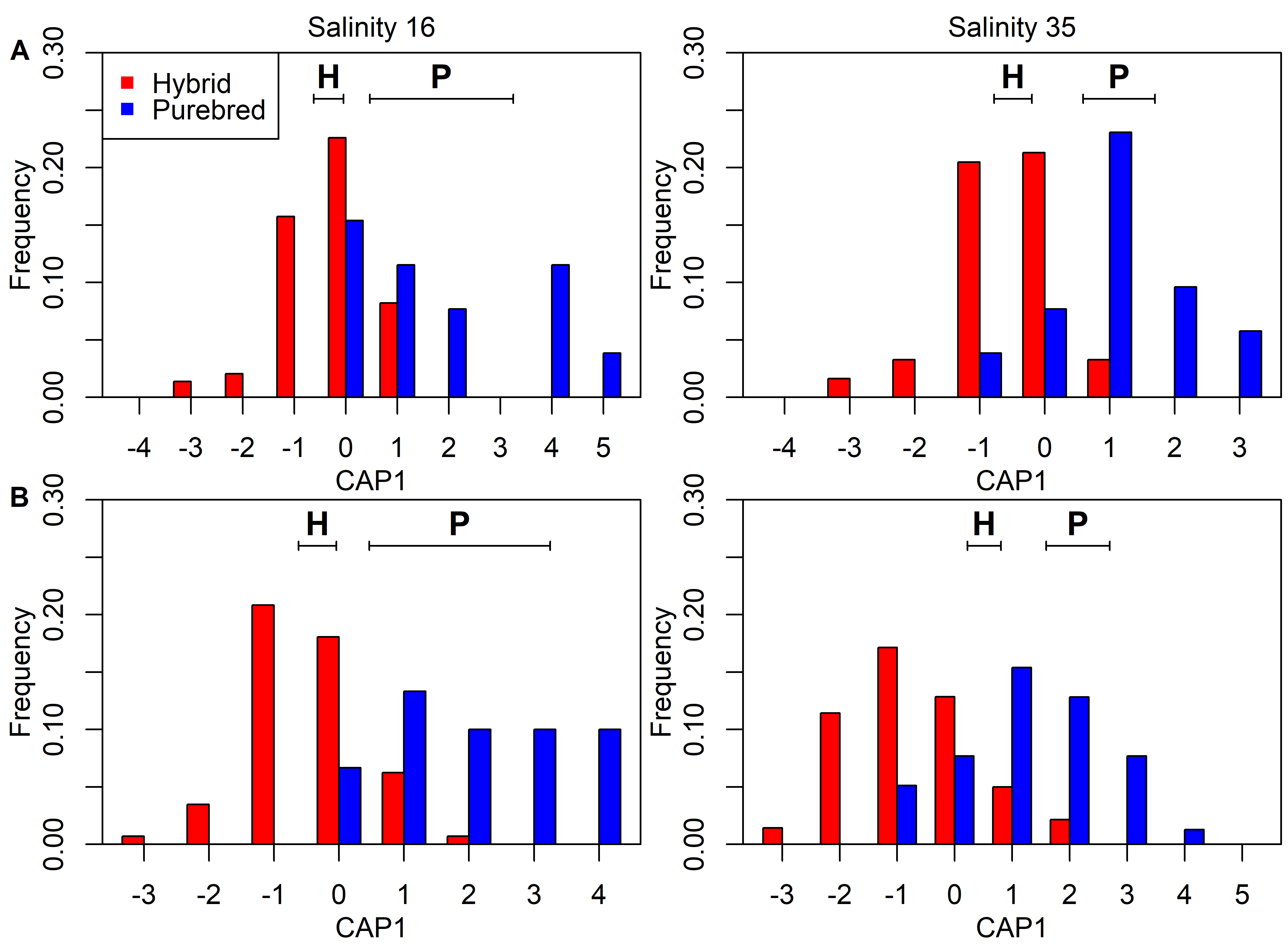

Supplement: S4 Fig — Data given for the samples A) 187 and B) 1098 days post hatching. Black bold letters represent the mean canonical value for each character ± 1*SE. Individual fish are represented by frequencies. (TIF) [file pone.0190995.s008.tif]

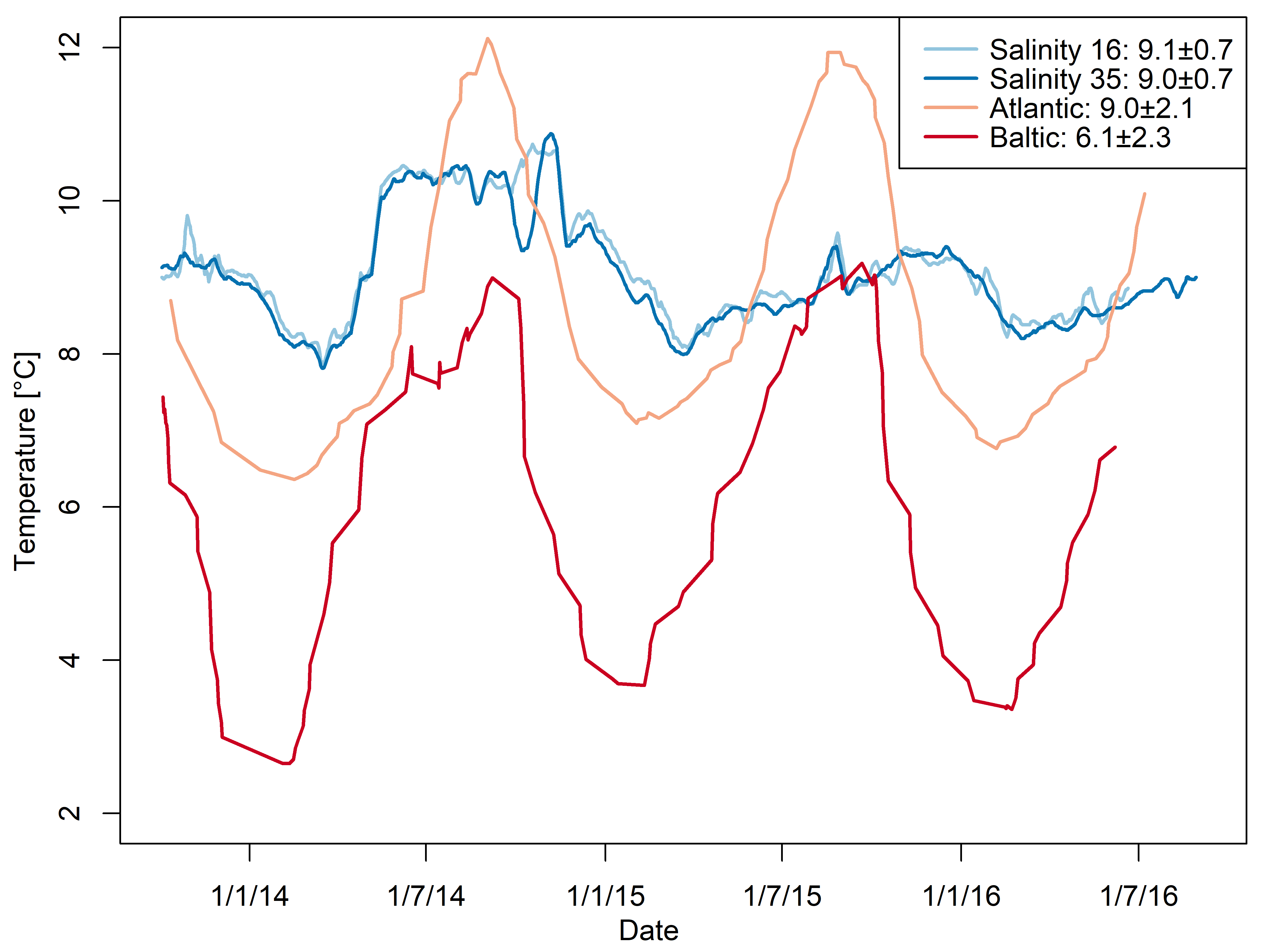

Supplement: S5 Fig — Water temperatures of the Atlantic (light red) were measured at stationary hydrographic stations in Ytre Utsira and Sognesjøen. Daily temperatures were combined for both stations and average for depths from 20–120 meters. Water temperatures of the Baltic (dark red) were extracted from https://sharkweb.smhi.se/ and restricted to the area 16–23° E and 56.5–62° N. Daily temperatures were combined all stations within the area and average for depths from 20–50 meters. Mean±SD are given in the legend and lines represent a running mean. (TIF) [file pone.0190995.s009.tif]

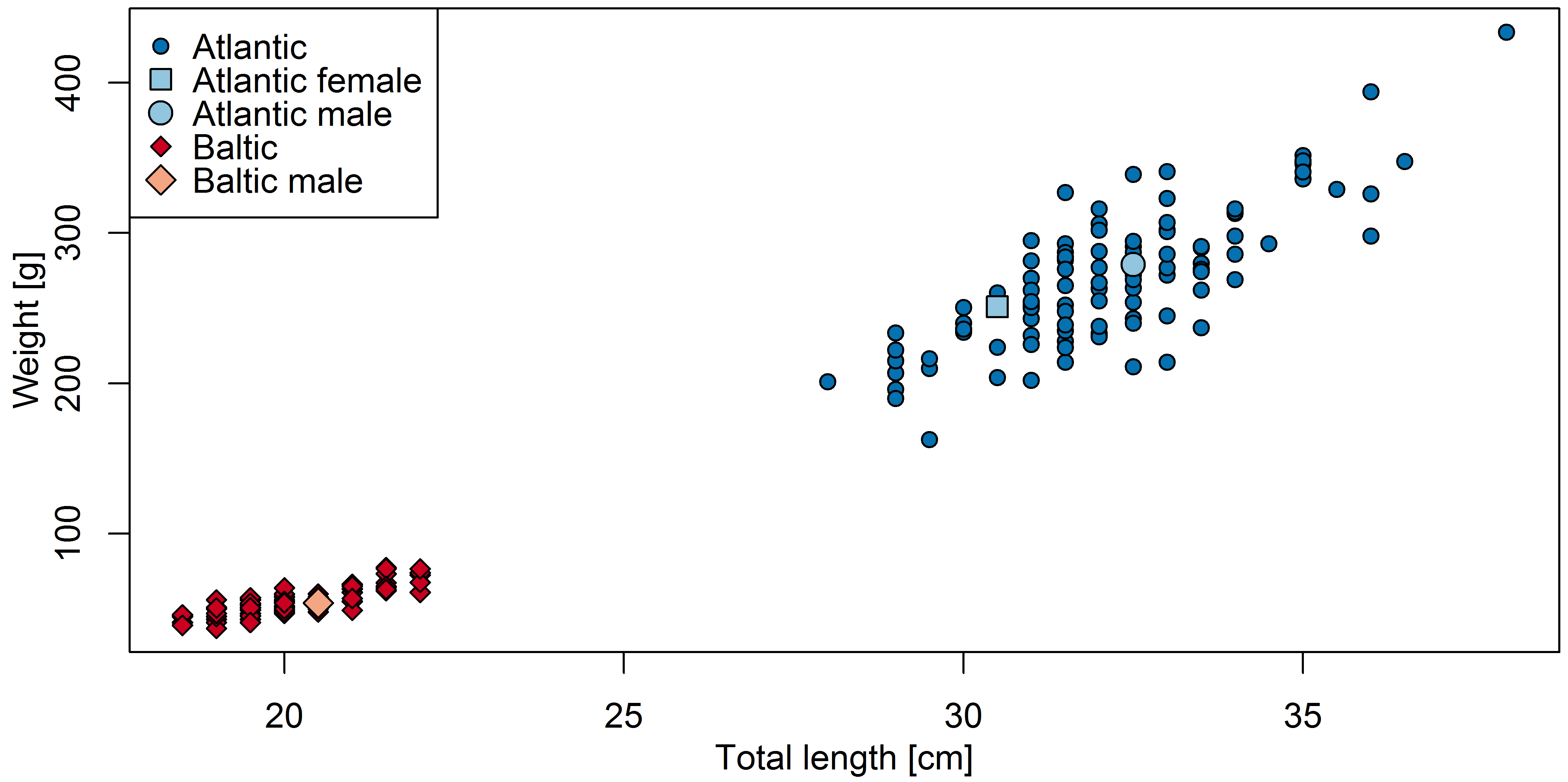

Supplement: S6 Fig — Individuals used as parents for the F1-generation are marked (Atlantic male, Atlantic female, Baltic male). (TIF) [file pone.0190995.s010.tif]

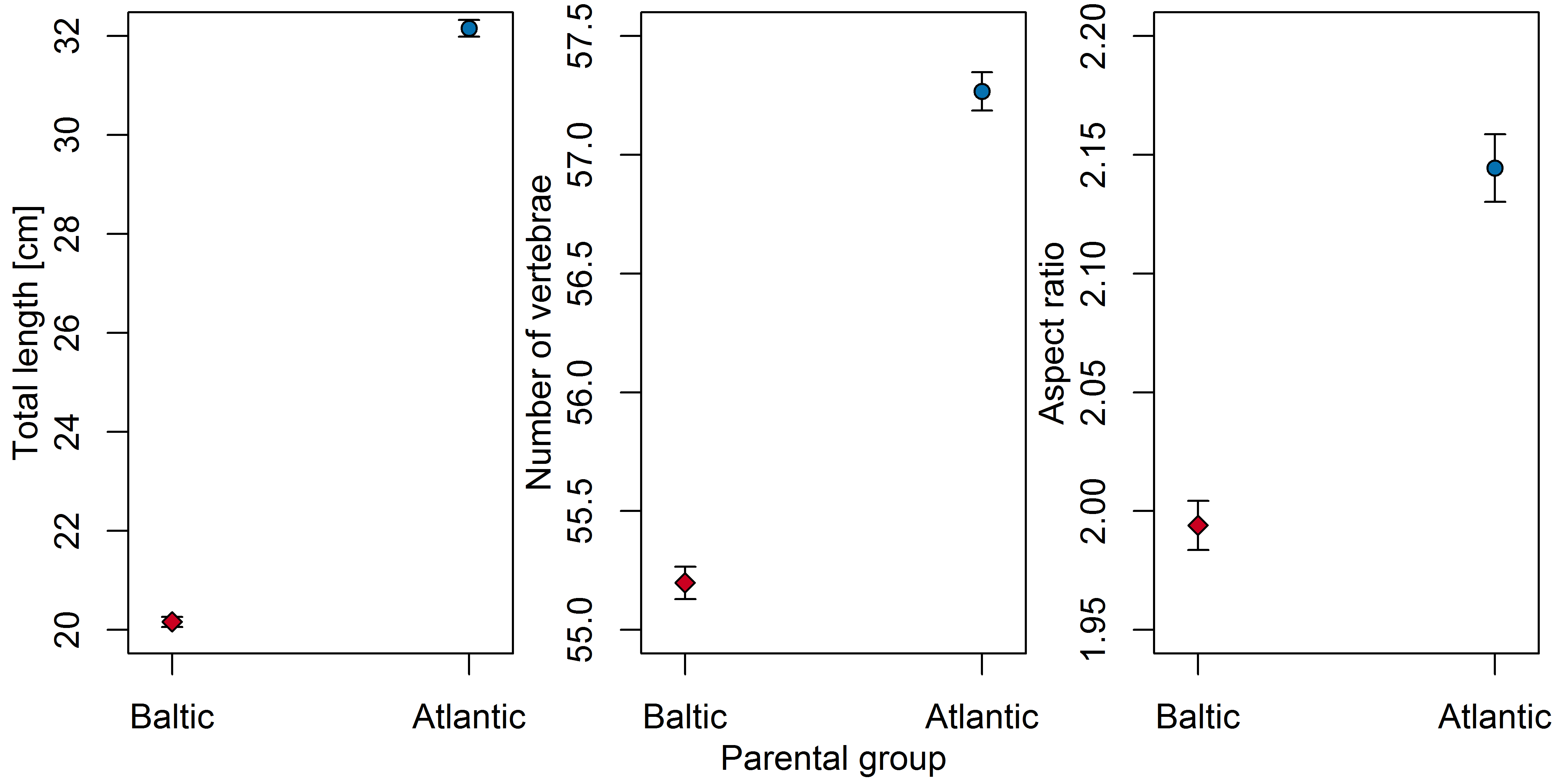

Supplement: S7 Fig — Mean values and 1*SE are shown. (TIF) [file pone.0190995.s011.tif]

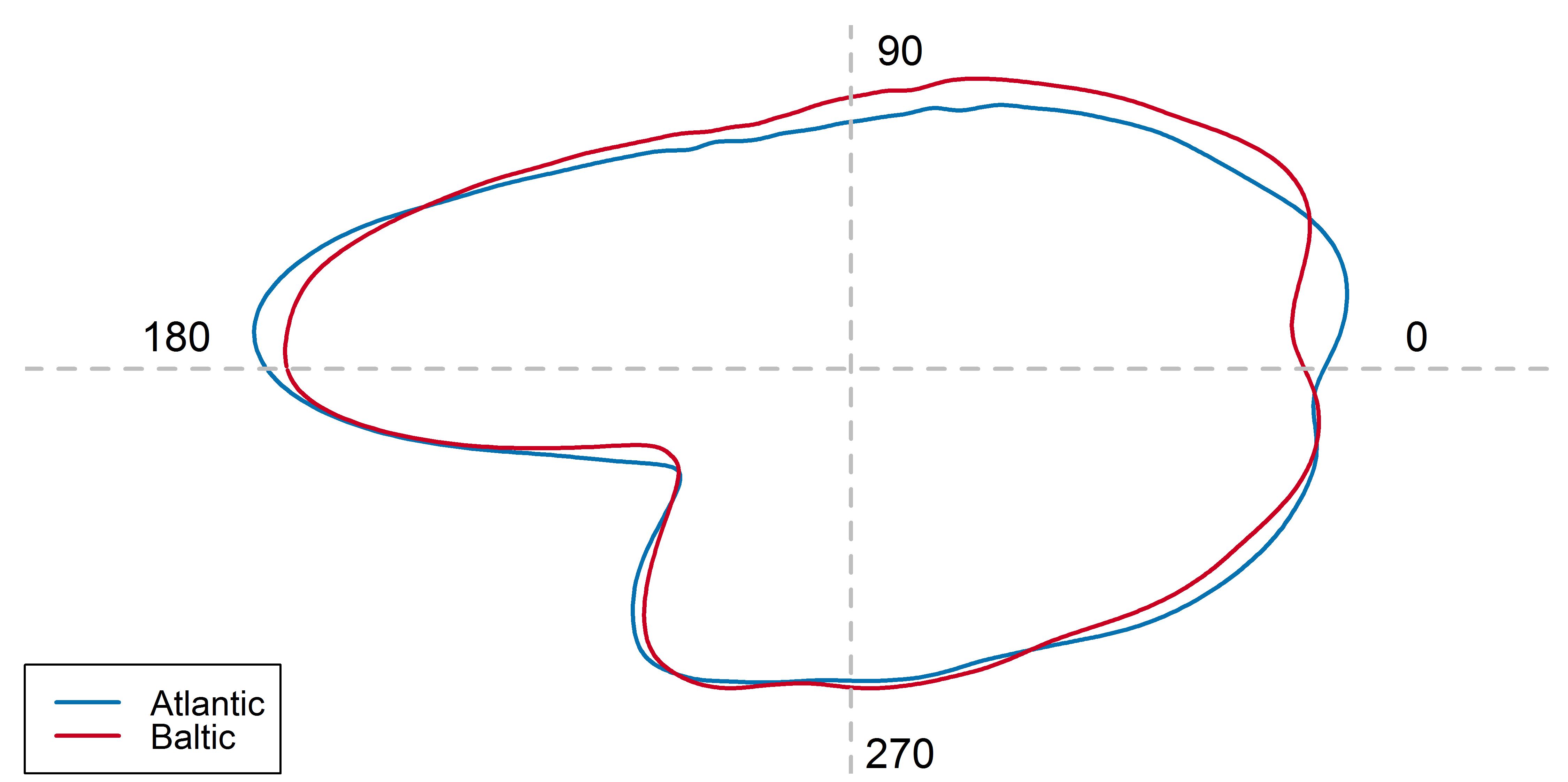

Supplement: S8 Fig — The shown outline does not correspond to the actual size and ratio of the original otoliths. (TIF) [file pone.0190995.s012.tif]
